# Smart Multi-Sensor Calibration of Low-Cost Particulate Matter Monitors

**DOI:** 10.3390/s23073776

**Published:** 2023-04-06

**Authors:** Edwin Villanueva, Soledad Espezua, George Castelar, Kyara Diaz, Erick Ingaroca

**Affiliations:** 1 Engineering Department, Pontificia Universidad Católica del Perú, 1801 Universitaria Av., San Miguel, Lima 15088, Peru; 2 Departamento Académico de Ingeniería, Universidad del Pacífico, 2020 Salaverry Av., Jesús María, Lima 15072, Peru; s.espezual@up.edu.pe; 3 Subgerencia de Gestión Ambiental, Municipalidad Metropolitana de Lima, Palacio Municipal, Lima 15001, Peru

**Keywords:** air quality, sensor calibration, multi-sensor calibration, low-cost sensor, machine learning, particulate matter

## Abstract

A variety of low-cost sensors have recently appeared to measure air quality, making it feasible to face the challenge of monitoring the air of large urban conglomerates at high spatial resolution. However, these sensors require a careful calibration process to ensure the quality of the data they provide, which frequently involves expensive and time-consuming field data collection campaigns with high-end instruments. In this paper, we propose machine-learning-based approaches to generate calibration models for new Particulate Matter (PM) sensors, leveraging available field data and models from existing sensors to facilitate rapid incorporation of the candidate sensor into the network and ensure the quality of its data. In a series of experiments with two sets of well-known PM sensor manufacturers, we found that one of our approaches can produce calibration models for new candidate PM sensors with as few as four days of field data, but with a performance close to the best calibration model adjusted with field data from periods ten times longer.

## 1. Introduction

According to estimates by the World Health Organization (WHO), air pollution causes seven million deaths yearly worldwide [1]. Epidemiological studies reveal that there is concrete evidence of the connection between poor air quality due to fine particulate matter (PM) and risk of chronic diseases [2,3,4]. Therefore, monitoring air quality within urban areas is a necessity for citizens, the health sector, and epidemiological and environmental research. Air quality information is also useful for informing local governments about the impact of public policies that mitigate air degradation. For example, to carry out interventions in sectors, such as energy, transport, waste management, agriculture, urban planning and sustainable economic development [5,6].

Traditionally, air quality information in urban areas is obtained through networks of high-end certified measurement stations, which provide data of guaranteed quality. However, the deployment of adequately sized monitoring networks of certified stations is often unfeasible for many cities due to their high acquisition and maintenance costs [7]. In response to this, a variety of low-cost sensor (LCS) technologies for assessing air quality have recently emerged. These solutions enable the deployment of large sensor networks, facing the challenge of monitoring air quality in extensive metropolises in real time and with high spatial resolution [5,8,9,10,11,12,13,14].

Despite these benefits, the reliability of the data captured by LCS technologies has frequently been questioned [5,8,10,15,16,17,18,19]. One approach commonly suggested by manufacturers to increase data quality is to adjust linear correction functions using calibration laboratories, where different concentrations of air pollutants are placed in a controlled manner, and the parameters of the calibration functions are estimated [7]. However, this method, in addition to being expensive because it involves the use of certified laboratories, assumes that the laboratory environment will be similar to the operating environment, which is often not the case in practice [17].

The other common approach to calibrate air quality sensors is called “*on-site* calibration” [15], in which a certified station (called “reference’) is co-located next to the LCS (called “candidate”), and simultaneous measurements are collected for a period of time. Then, a correction function is adjusted to approximate the measurements of the candidate sensor to that measured by the reference sensor [5,18,19]. On-site calibration is often preferred over laboratory calibration because calibration functions fitted with data collected from the environment in which the sensor will operate are expected to better represent reality [15]. However, building a proficient calibration function is always a challenge, since many factors affect the sensor’s response, such us meteorological conditions, pollution conditions, particle composition and sizes, sensor aging, among others [15]. In particular, one area of challenge is when conditions go outside a previously “seen” range. Most of the works on sensor calibration for air quality assessment are aimed at per-sensor calibration functions due to the variability of sensor behaviors, even if they are of the same type, manufacturer and environmental conditions [6,14,16,20,21].

Among the most popular calibration function types are simple linear regression (use only the candidate sensor measurement as input variable) and multivariate linear regression (incorporate additional input variables to the regression, such as temperature, humidity, etc.) [18,22]. However, limitations have been found in these approaches because they fail to follow the nonlinearities of the sensors and the complex atmospheric processes that influence them [5,23]. For example, the following factors are sources of error for the correction functions: sensor behavior change due to age, sensor dynamic limits, and weather and pollution conditions where the sensor is located [5,24,25].

Machine learning (ML) methods have shown promise in dealing with sensor nonlinearities and exploiting different local and context variables [5,6,7,9,14,18,26,27]. Most of these proposals are focused on the adjustment of ad hoc calibration functions for the candidate sensor. This implies that for each new candidate sensor that is to be installed in the monitoring network, the respective correction function must be estimated, implying the need to collect *on-site* data with a reference station and the costs and time that this implies.

In this paper, we describe and evaluate ML-based approaches to calibrate LCS of particulate matter (PM) for which we do not have on-site data against a reference station (or we have a limited amount of these data) but for which we have field data from other sensors (base sensors) of the same manufacturer that passed an inter-comparison campaign with a reference station in the same city. The aim is to leverage existing field data in the process of building calibration models for new sensors and thus facilitate their rapid incorporation into the monitoring network and ensure the quality of the data they provide.

We studied different ML algorithms and different multi-sensor calibration strategies in order to identify the most suitable ones for the described problem. A comprehensive experimental evaluation was performed using data from two sets of sensors from well-known manufacturers deployed in Lima city. The rest of the paper is organized as follows. Section 2 describes the air quality devices, the field data collection, and the methods used for building the calibration model. Section 3 describes the experimental setup, results and discussion. Finally, Section 4 presents the conclusion and delineates future research that the present work can generate.

## 2. Materials and Methods

### 2.1. Sensing Devices

In this study, we evaluated two sets of LCS from two different manufactures: IQAir and AirBeam, which are described next.

#### 2.1.1. IQAir AirVisual Devices

AirVisual devices are manufactured by IQAir company. This is one of the world’s largest providers of low-cost air quality measurement solutions. It also maintains a web platform (AirVisual) that displays air quality information from thousands of monitoring stations around the world. In this study, we evaluated four IQAir devices as candidate sensors to be calibrated. These devices possess light-scattering laser photo sensors capable of measuring airborne particles ($PM_{2.5}$ and $PM_{10}$) along with temperature and relative humidity (RH) in real-time concentrations. The particulate matter (PM) sensors have a measuring range of 0–1000 μg/m${}^{3}$ (resolution of 0.1 μg/m${}^{3}$). The temperature sensor has a measuring range of −30 to 60 ${}^{\circ }$C (resolution of 0.1 ${}^{\circ }$C). The humidity sensor has a measuring range of 0 to 100% RH (non-condensing) with a resolution of 1%. The devices have a local storage capacity and also Wi-Fi connectivity for continuous data sharing. According to the manufacturer’s website, the sensors are calibrated at the factory to ensure high precision. In what follows, IQAir sensors will be identified with the initials HC.

#### 2.1.2. AirBeam Devices

AirBeam is a small low-cost monitor manufactured by HabitatMap company (www.habitatmap.org). In this study, we evaluated three AirBeam devices as candidate sensors to be calibrated. The device possesses a digital universal particle concentration sensor (Plantower PMS7003) to measure concentrations of airborne particles ($PM_{2.5}$ and $PM_{10}$) along with temperature and humidity sensors. The PM sensors have a measuring range of 0–1000 μg/m${}^{3}$ (resolution of 1 μg/m${}^{3}$). The temperature sensor has a measuring range of −40 to 150 ${}^{\circ }$C (resolution of 1 ${}^{\circ }$F). The humidity sensor has a measuring range of 0 to 100% RH (non-condensing) with a resolution of 1%. The devices have a local storage capacity and also Wi-Fi connectivity for continuous data sharing. In what follows, AirBeam sensors will be identified with the initials AB.

#### 2.1.3. Reference Air Quality Station

The reference station used in this study was a Teledyne API T640 Mass Monitor. This instrument is a federal equivalent method (FEM), as designed by the U.S. Environmental Protection Agency (EPA). The device uses scattered light spectrometry for measurement and has the capability to continuously measure $PM_{2.5}$ and $PM_{10}$ concentrations. The measuring range is 0–10,000 μg/m${}^{3}$ with a resolution of 0.1 μg/m${}^{3}$ and 1-h average precision of ±0.5 μg/m${}^{3}$. The equipment is operated by the Municipalidad Metropolitana de Lima.

### 2.2. Field Data Collection

**Figure 1:**
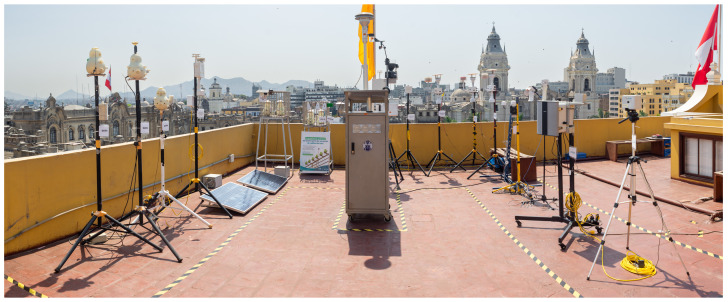
Place of the data collection campaign and the arrangement of the sensors. The reference station (Teledyne) is located in the center.

**Figure 2:**
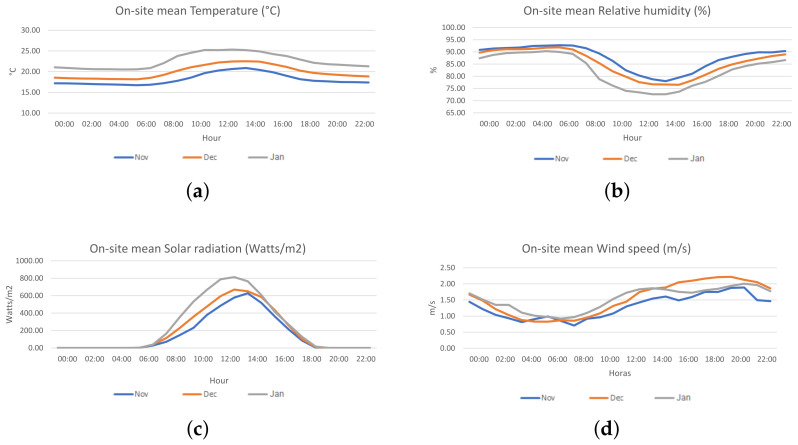
Plots of hourly mean temperature (**a**), relative humidity (**b**), solar radiation (**c**) and wind speed (**d**) for the three months involved in the study (November 2021 to January 2022). The hourly mean temperature and solar radiation increase with the arrival of summer (December–March), while the humidity decreases.

A field data collection campaign was conducted with the above instruments between the months of November 2021 to January 2022 in the city of Lima (Peru). The place of the campaign was the roof of the Municipal Palace of Lima, located at the Lima main square at latitude −12.045287317106624, longitude −77.03090612125114 (UTM Easting 278922, UTM Northing 8667635) and altitude of 160 m above sea level. The height of the building was about 8 m. Figure 1 shows the arrangement of the evaluated devices, located at a maximum distance of 2 m from the reference station. The meteorological conditions of the site in the period of data collection correspond to the end of the spring season and the beginning of summer, with temperatures ranging from 18 to 26 ${}^{\circ }$C and relative humidity between 70% and 90%. The hourly averages of the wind speed vary between 0.5 and 2.1 m/s, with the lowest values appearing between 5 and 9 h and the highest values between 17 and 21 h. The prevailing wind direction is from the south, followed by the southeast. Figure 2a–d shows plots of hourly averages of temperature, humidity, solar radiation, and wind speed, respectively, throughout the day for each month involved in the data collection campaign.

### 2.3. Data Analysis

The time resolution of the raw data collected by the IQAir devices was fifteen minutes. The time resolution of the raw data collected by the AirBeam devices was one minute. The time resolution of the reference station was hourly. Therefore, we converted the data of all devices to an hourly frequency (using mean aggregation) to be comparable. The periods of field data collection for each set of sensors were as follows:

- IQAir: from 24 November 2021 to 30 December 2021 (873 h);
- AirBeam: from 15 November 2021 to 8 January 2022 (1320 h).

For purposes of development and evaluation of calibration models, the data were divided into three time periods: Train (training), Ack (acknowledge) and Test (testing). Data from Train and Ack periods were used to build the models, as will be described in the next section. Data from the Test period were exclusively used for testing the performance of the developed models.

**Figure 3:**
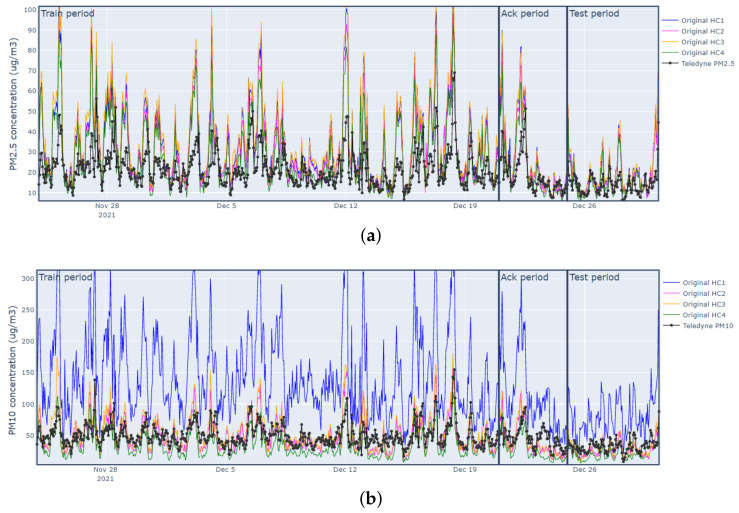
Plots of $PM_{2.5}$ and $PM_{10}$ hourly data collected by IQAir sensors and reference sensor (Teledyne) during 24 November 2021 to 30 December 2021: (**a**) hourly $PM_{2.5}$ series measured by IQAir sensors; (**b**) hourly $PM_{10}$ series measured by IQAir sensors.

Figure 3 shows the hourly time series of $PM_{2.5}$ and $PM_{10}$ values registered by the IQAir sensors (denoted as HC1, HC2, HC3, HC4) and the reference (Teledyne) sensor in the considered period. We saw a noticeable overestimation of the $PM_{2.5}$ IQAir sensors when compared to the reference instrument, specially when the pollutant concentrations were high. As for $PM_{10}$, we observed a significant overestimation in the HC1 sensor, although apparently the other $PM_{10}$ sensors were close to the reference.

**Figure 4:**
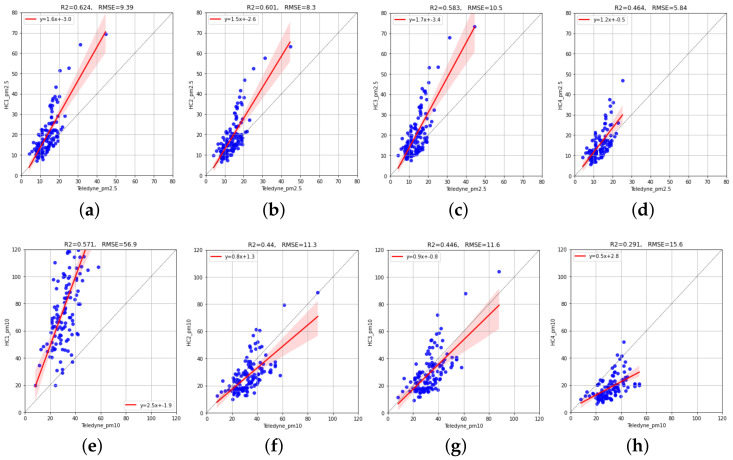
Scatterplots of the reference (Teledyne) vs. IQAir hourly measurements in the Test period. The top row shows results for the $PM_{2.5}$ sensors. The bottom row shows results for the $PM_{10}$ sensors. The red line represents the linear regression function between candidate sensor values and reference sensor values adjusted with data from the Train period: (**a**) HC1 ($PM_{2.5}$); (**b**) HC2 ($PM_{2.5}$); (**c**) HC3 ($PM_{2.5}$); (**d**) HC4 ($PM_{2.5}$); (**e**) HC1 ($PM_{10}$); (**f**) HC2 ($PM_{10}$); (**g**) HC3 ($PM_{10}$); (**h**) HC4 ($PM_{10}$).

To have a clearer idea of this, Figure 4 shows scatterplots of reference (Teledyne) vs. IQAir hourly values in the Test period. We can verify the overestimation bias of $PM_{2.5}$ sensors (regression lines above the diagonal line) and a moderate degree of accompaniment (coefficient of determination $R^{2}$ between 0.46 and 0.62). Regarding the $PM_{10}$ sensors, we can verify the large overestimation of the HC1 sensor, although this has the best $R^{2}$ of all $PM_{10}$ sensors (0.57). The other sensors do not show an overestimation bias (in fact, they underestimate), but they present low accompaniment ($R^{2}$ less than 0.44).

**Figure 5:**
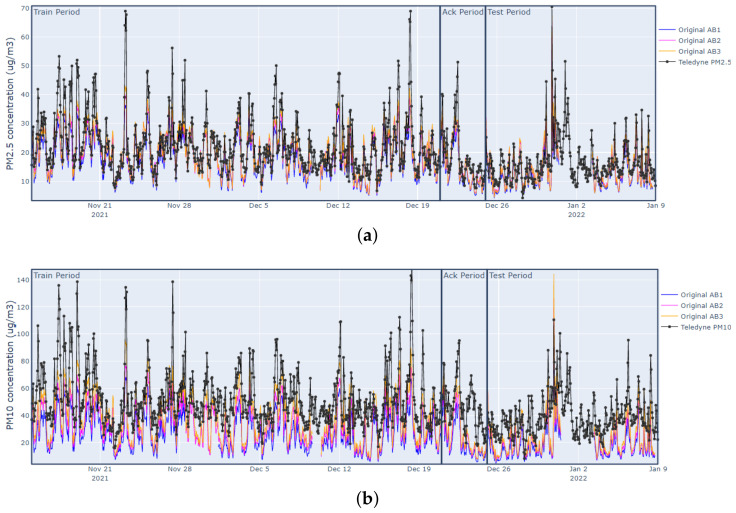
Plots of $PM_{2.5}$ and $PM_{10}$ hourly data collected by the AirBeam sensors and the reference sensor (Teledyne) during 15 November 2021 to 8 January 2021: (**a**) hourly $PM_{2.5}$ series measured by the AirBeam sensors; (**b**) hourly $PM_{10}$ series measured by the AirBeam sensors.

Figure 5 shows the hourly time series of $PM_{2.5}$ and $PM_{10}$ values registered by the AirBeam sensors (denoted as AB1, AB2, AB3) and the reference sensor (Teledyne) in the studied period.

**Figure 6:**
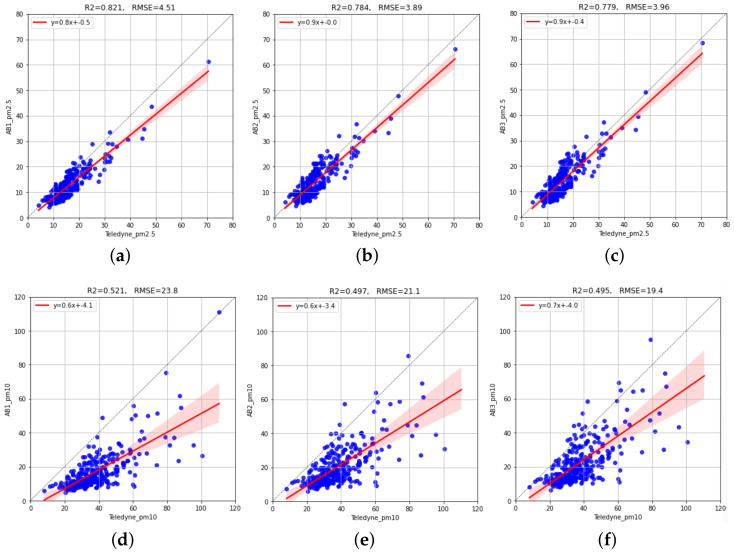
Scatterplots of the reference (Teledyne) vs. AirBeam sensor measurements in the Test period. The top row shows results for $PM_{2.5}$ sensors. The bottom row shows results for $PM_{10}$ sensors. The red line represents the linear regression function between candidate sensor values and reference sensor values adjusted with data from the Train period: (**a**) AB1 ($PM_{2.5}$); (**b**) AB2 ($PM_{2.5}$); (**c**) AB3 ($PM_{2.5}$); (**d**) AB1 ($PM_{10}$); (**e**) AB2 ($PM_{10}$); (**f**) AB3 ($PM_{10}$).

Figure 6 shows scatterplots of the reference (Teledyne) vs. AirBeam values in the Test period. We can see that $PM_{2.5}$ sensors do not present significant measurement bias (the regression lines are close to the diagonal). In addition, they present a good accompaniment, with $R^{2}$ between 0.78 and 0.82. With respect to the $PM_{10}$ sensors, these exhibit a marked underestimation bias and a moderate accompaniment ($R^{2}$ around 0.5).

### 2.4. Calibration Models

Two kinds of calibration models were evaluated: monosensor and multisensor calibration.

#### 2.4.1. Monosensor Calibration Models

This type of model is the most common in the literature. In this approach, the correction model is adjusted for a specific candidate sensor using data from a period of simultaneous measurements of that sensor and a co-located reference sensor. For better explanation, we will assume that we have organized such data into a set of *n* observation instances $D={\left\{\left(x_{i},y_{i}\right)\right\}}_{i=1}^{n}$. Each instance $\left(x_{i},y_{i}\right)$ is formed by an input feature vector obtained at time *i*, $x_{i}\in X$, and the reference (target) value $y_{i}\in Y$ obtained at the same time. In general, the input feature variables X are composed of the PM measurement of the candidate device and other variables that it can measure and that can help in the correction (temperature, humidity, etc.). The modeling process involves finding a mapping function $f_{\theta }:X\to Y$ (the model) with parameters θ that minimize at each observation instance $\left(x_{i},y_{i}\right)$ some loss function $l(\hat{y_{i}},y_{i})$ that expresses the divergence between the predicted value $\hat{y_{i}}=f_{\theta }\left(x_{i}\right)$ and the actual reference value $y_{i}$. For this work, we use the common squared error loss $l\left(\hat{y_{i}},y_{i}\right)={\left(\hat{y_{i}}-y_{i}\right)}^{2}$. With this, the empirical loss of the model $f_{\theta }$ on the whole training set *D* is the mean squared error (MSE), defined as Equation (1).

(1) $$MSE_{D}\left(f_{\theta }\right)=\frac{1}{n}\sum_{(x_{i},y_{i})\in D}{\left(f_{\theta }\left(x_{i}\right)-y_{i}\right)}^{2}$$

For the present study, we evaluated the machine learning (ML) methods listed below as inductors of monosensor calibration models. As input variables (features), we considered the common ambient parameters sensed by the candidate devices (particulate matter concentration (PM), temperature (T) and relative humidity (RH)); thus, a data instance is a vector $x_{i}=\left[PM_{i},T_{i},RH_{i}\right]$. For the implementation and experimentation of the ML methods, we used Python language and the library scikit-learn (Sklearn).

- **Univariate Linear Regression**: The calibration model follows the form: $f_{\theta }\left(x_{i}\right)=\theta_{0}+\theta_{1}PM_{i}$. The parameters are optimized using the ordinary least squares technique implemented in the Sklearn library.
- **lMultivariate Linear Regression (MLR)**: The calibration model follows the form [28]: $f_{\theta }\left(x_{i}\right)=\theta_{0}+\theta_{1}PM_{i}+\theta_{2}T_{i}+\theta_{3}RH_{i}$. The parameters are optimized using the ordinary least squares technique implemented in the Sklearn library.
- **Nearest Neighbors Regression (KNN)**: This model predicts the target value of a testing data instance based on the local interpolation of the targets of the *k*-nearest neighbors instances in the training set. As distance metric, we use the Euclidean distance and five neighbors, which experimentally showed to be adequate. Hagan et al. [29] applied this approach for SO_2_ sensor calibration with good results.
- **Support Vector Regression (SVR)**: This model is the extension of support vector machines (SVM) for regression tasks. Only a subset of training instances is used in the model (the support vectors), ignoring instances whose prediction is close to the target. For the present study, we used default hyperparameters of the SVR implementation in the Sklearn library: linear kernel, regularization parameter C = 1, maximum number of iterations = 1000. Bigi et al. [30] applied SVR for NO/NO_2_ sensor calibration with better results than linear models.
- **AdaBoost Regression**: This method sequentially builds an ensemble of predictors. Each predictor is fitted using a weighted version of the dataset, where weights are adjusted according to the error of the last predictor; thus, each new predictor focuses on more difficult cases. In this study, we used default hyperparameters of AdaBoost implementation in Sklearn: number of predictors = 100 and learning rate = 1.0. Liang et al. [31] applied this method to predict air quality index (AQI) levels with outstanding results among various ML methods.
- **Gradient Boosting Regression (GB)**: This method additively builds an ensemble of predictors (decision trees). Each predictor is fit on the negative gradient of the loss function of the previous model, thus trying to correct its errors. In this study, we used default hyperparameters of AdaBoost implementation in Sklearn: number of predictors = 100, learning rate = 0.1, maximum depth of individual predictors (max_depth) = 3. Johnson et al. [32] applied this approach to calibrate $PM_{2.5}$ sensors, finding a better performance than linear models.
- **Random Forest Regression (RF)**: This is an ensemble method that builds several decision trees by sampling the training set with replacement (bagging) and also performs feature sampling (random subspace). The final prediction is the average of individual predictors, thus avoiding overfitting. In our experimentation, we used default hyperparameters of random forest in Sklearn: number of predictors = 100, max_depth = none (nodes expanded until all leaves are pure or contain two samples). RF has been widely used in air quality sensor calibration with promising results [33,34].
- **Extra Trees Regression (ET)**: This method is similar to RF in that multiple decision trees are fitted to random sub-samples of the training set, and their predictions are mean aggregated at testing stage. However, the sub-sampling is performed without replacement. In addition, the nodes of decision trees are split randomly instead of using a purity criterion. In our experiments, we used default hyperparameters of random forest in Sklearn: number of predictors = 100, max_depth = none (nodes expanded until all leaves are pure or contain two samples). This method has shown outstanding performance predicting AQI values [35].

#### 2.4.2. Multisensor Calibration Models

This type of model is proposed here to calibrate a new sensor $S_{T}$ (target sensor) to be incorporated into an existing sensor network. We assume that the sensors in operation (base sensors) have passed an inter-comparison campaign with a reference station. However, we consider that we do not have such field data for the new target sensor (or we have a limited amount of these data).

Let us denote the set of base sensors as $S_{1},S_{2},\ldots S_{k}$ and their corresponding field datasets as $D_{1},D_{2},\ldots D_{k}$. Each dataset $D_{i}$, called the base dataset, has a similar structure as described in the previous section, namely: $D_{i}={\left\{\left(x_{i},y_{i}\right)\right\}}_{i=1}^{ni}$. With dataset $D_{i}$, we can induce a calibration model $f_{i}\left(\right)$ for the base sensor $S_{i}$ using any of the ML methods described in the previous section. Let us denote the set of resulting calibration models (called base models) as $\left\{f_{1},f_{2},\ldots ,f_{k}\right\}$. We drop θ from the model’s notation for simplicity, but it is understood that each model is defined by its fitted parameters θ.

**Figure 7:**
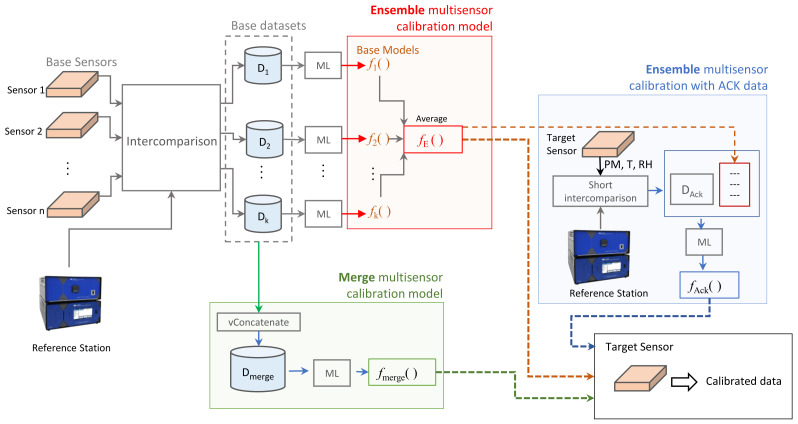
Proposed framework for multisensor calibration modeling.

In Figure 7, we present a modeling framework from which we can derive various approaches to calibrate the new target sensor $S_{T}$ based on the available base datasets or base models. Next, we describe calibration models derived from this framework.

- **Merge Multisensor Calibration Model**: This model is obtained by first merging the base datasets (vertical concatenation): $D_{merge}=vConcatenate\left(D_{1},D_{2},\ldots D_{k}\right)$ and then fitting a single model $f_{merge}$ on this merged dataset using the same ML methods as those indicated in Section 2.4.1. The idea behind fitting a model with data from several base sensors is that it can generalize the calibration task to new sensors.
- **Ensemble Multisensor Calibration**: This model uses the base models to correct the PM measurement of the target sensor at time point *i* as the mean value of the predictions of the base models given the input feature vector $x_{i}$ obtained at that time *i* by the target sensor (Equation (2))
  (2) $$\hat{y_{i}}=f_{E}\left(x_{i}\right)=\frac{1}{k}\sum_{j=1}^{k}\left(f_{j}\left(x_{i}\right)\right)\;$$
- **Ensemble Multisensor Calibration with Acknowledge Period**: In this approach, we assume that the target sensor passed a short intercomparison period with a reference station (called the acknowledge period) obtaining a dataset $D_{Ack}={\left\{\left(x_{i},y_{i}\right)\right\}}_{i=1}^{n_{ack}}$. Then, this dataset is column-augmented by the predictions of the above ensemble multisensor calibration (Equation (2)). This means that the extended dataset has the form ${\tilde{D}}_{Ack}={\left\{\left(\left[x_{i},f_{E}\left(x_{i}\right)\right],y_{i}\right)\right\}}_{i=1}^{n_{ack}}$. With this dataset, we propose to fit the final calibration model for the target sensor $f_{Ack}\left(x_{i}\right)$ using an ML method. The idea of having as input variable the predictions of the ensemble of base models is to take advantage of the knowledge captured on them, since they were fitted to a significant greater amount of field data. In this way, it is expected to make efficient use of all available data and, at the same time, to customize the model to the target sensor to improve efficiency in calibrating it.

## 3. Results and Discussion

### 3.1. Performance Evaluation of Monosensor Calibration Models

We first assessed the performance of the monosensor approaches described in Section 2.4.1. For each sensing device and ML method, we performed a 10-fold cross-validation evaluation on the training set (see Section 2.3) in order to determine the method that best induces monosensor calibration models. More precisely, for a given sensor $S_{i}$, we randomly split the corresponding training set $D_{i}^{trn}$ into 10 equal parts (folds). Then, in an iterative way, one fold is separated for testing and the remaining folds are used for fitting the model, which is asked to predict the targets of the testing fold, and then an error metric is calculated. After evaluating each fold as a test set, the mean and standard deviation of the error metrics are calculated. We used the square root of the MSE (*RMSE*) as an error metric, which can be interpreted in the same units of the predicted variable. All models and evaluation were implemented in Python programming language using the Scikit-learn ML library. For the ensemble methods, we used 500 estimators (it was verified experimentally that more than this value did not improve the results).

Table 1 and Table 2 show the cross-validation error metrics and standard deviations (in parenthesis) for $PM_{2.5}$ and $PM_{10}$, respectively. In general, we can see that among the methods with less performance are the SVR and the multivariate linear regression. The methods with the best results are random forest and extra trees. These methods show very close results, although with extra trees having some tendency to overcome random forest in $PM_{2.5}$. In $PM_{10}$, both methods offer remarkable results, without a clear trend of which is better. Interestingly, the KNN method presents results close to the best despite its simplicity, although with a higher standard deviation, which can represent unwanted performance variability with small perturbation to the training and test data. These results are in line with other results reported in the literature relating appealing results of ensemble methods in calibrating air quality sensors [32,33,34,36,37], confirming the nonlinear behavior of these devices.

### 3.2. Performance Evaluation of Multisensor Calibration Models

The above evaluation showed that the random forest and extra trees methods consistently induce models with the best cross-validated performances on the training set, with a slight advantage for the extra trees method. Because of this, we selected this method to induce the base models for the multisensor calibration approaches. The three approaches (Section 2.4.2) were evaluated on a per-sensor cross-validation strategy: for given manufacturer (IQAir or AIRBeam), one sensor was chosen as the candidate sensor to be calibrated (its dataset was separated for testing the models). The remaining sensor’s datasets were used to induce the multisensor calibration model, which was then asked to predict the calibrated test measurements of the candidate sensor. Then, performance indices were calculated. This process was repeated until every sensor was evaluated as a candidate sensor. As performance indices, we used the coefficient of determination ($R^{2}$) and the *RMSE* index. The $R^{2}$ index measures the proportion of co-variation between the model’s predictions and the reference values. A value of one means a perfect accompaniment. A value less than or equal to zero means that the reference’s mean is a better prediction than what the fitted model predicts. The *RMSE* is a scale-dependent index and measures the actual differences between the model predictions and the reference values. Both indices give a different and complementary perspective of a model’s performance. For each candidate sensor and calibration approach, we ran ten independent evaluations to obtain ten performance metrics. Then, for each candidate sensor, we compared the means of the performance values among all pairs of studied calibration methods with a t-test statistical test. We found that all pairs of calibration methods show statistical differences in the means of performance metrics under the 0.05 significance level.

**Figure 8:**
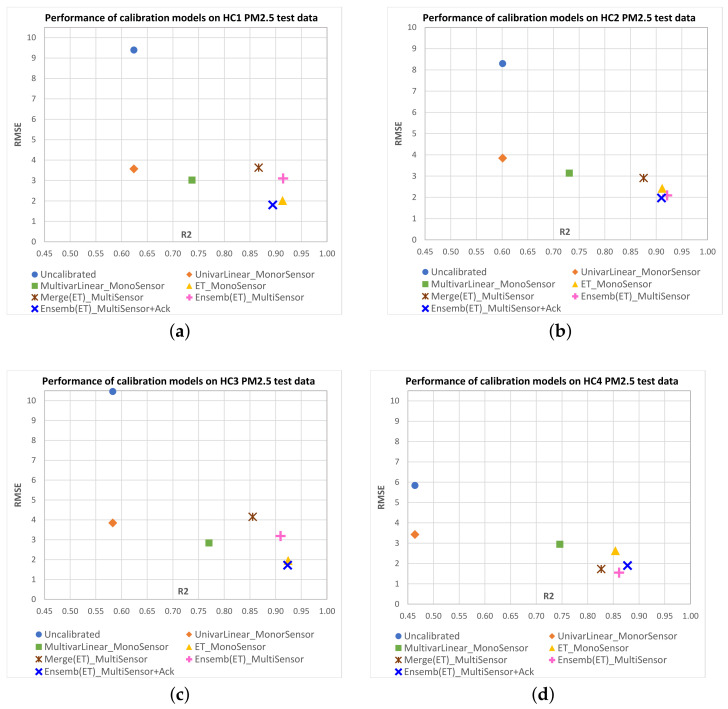
Scatterplots of $R^{2}$ vs. RMSE mean values obtained on test data for each IQAir $PM_{2.5}$ sensor as candidate sensor with the different calibration models. The ideal performance is the point with RMSE=0 and $R^{2}=1$, which is the lower right corner of the plots. Points closest to that corner represent models with better performance. Subfigures correspond to the following $PM_{2.5}$ candidate sensors: (**a**) HC1; (**b**) HC2; (**c**) HC3; (**d**) HC4.

**Figure 9:**
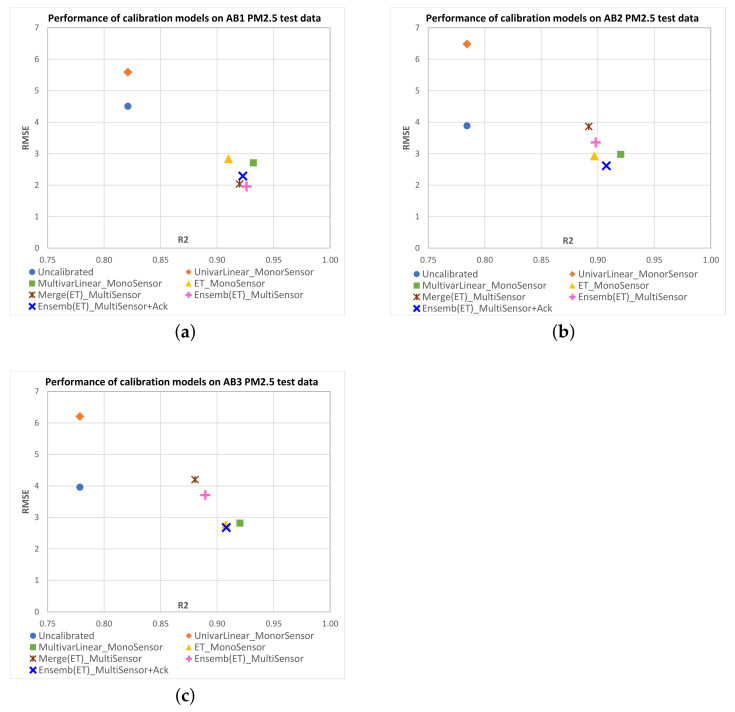
Scatterplots of $R^{2}$ vs. RMSE mean values obtained on test data for each AIRBEAM $PM_{2.5}$ sensor as candidate sensor with the different calibration models. The ideal performance is the point with RMSE=0 and $R^{2}=1$, which is the lower right corner of the plots. Points closest to that corner represent models with better performance. Subfigures correspond to the following $PM_{2.5}$ candidate sensors: (**a**) AB1; (**b**) AB2; (**c**) AB3.

**Figure 10:**
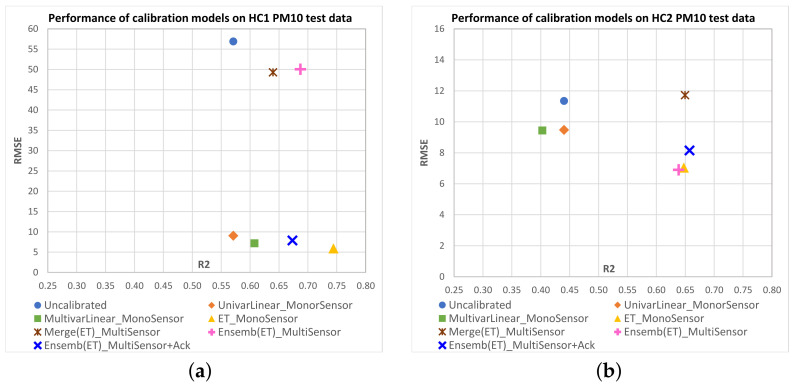

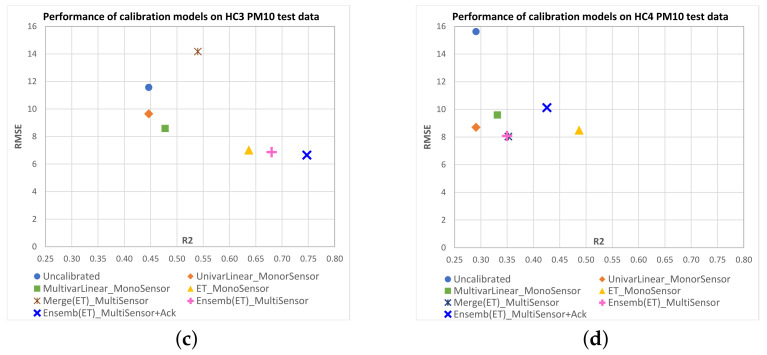
Scatterplots of $R^{2}$ vs. RMSE mean values obtained on test data for each IQAir $PM_{10}$ sensor as candidate sensor with the different calibration models. The ideal performance is the point with RMSE=0 and $R^{2}=1$, which is the lower right corner of the plots. Points closest to that corner represent models with better performance. Subfigures correspond to the following $PM_{10}$ candidate sensors: (**a**) HC1; (**b**) HC2; (**c**) HC3; (**d**) HC4.

**Figure 11:**
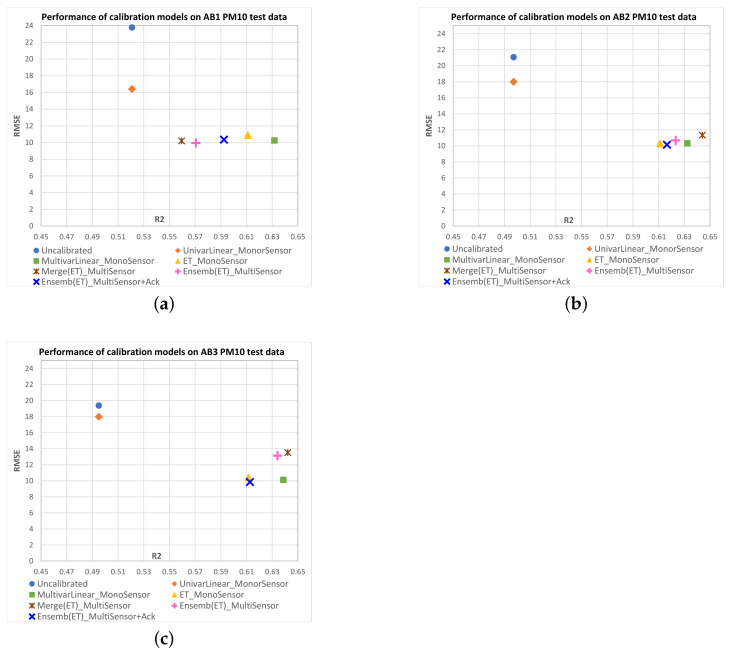
Scatterplots of $R^{2}$ vs. RMSE mean values obtained on test data for each AIRBEAM $PM_{10}$ sensor as candidate sensor with the different calibration models. The ideal performance is the point with RMSE=0 and $R^{2}=1$, which is the lower right corner of the plots. Points closest to that corner represent models with better performance. Subfigures correspond to the following $PM_{10}$ candidate sensors: (**a**) AB1; (**b**) AB2; (**c**) AB3.

Figure 8 shows scatterplots of $R^{2}$ vs. RMSE mean values obtained on the test data for each IQAir $PM_{2.5}$ target sensor with the different multisensor approaches. Figure 9 shows equivalent results for AirBeam $PM_{2.5}$ sensors, and Figure 10 and Figure 11 show results for $PM_{10}$ sensors. Additionally, we have included in the plots the mean performance points of original measurements (uncalibrated) and the monosensor models: univariate linear regression (UnivarLinear_MonoSensor), multivariate linear regression (MultivarLinear_MonoSensor) and extra trees regression (ET_MonoSensor), which was the best monosensor model. The ideal performance is when RMSE=0 and $R^{2}=1$, which corresponds to the lower right corner of the plots. Thus, points closest to that corner represent models with better performance.

We can see that in the case of IQAir sensors, uncalibrated measurements perform worse than calibrated data by any model. The UnivarLinear_MonoSensor model noticeably improves the RMSE index on IQAir sensors, but the $R^{2}$ remains unchanged as expected. In the case of AirBeam, the UnivarLinear_MonoSensor correction worsens the RMSE with respect to the uncalibrated data. In all sensors, the corrections performed with multivariate models present better RMSE and $R^{2}$ indices than the uncalibrated data or calibrated data with univariate models. This effect can be attributed to the temperature and humidity input variables in the multivariate models, which can be deduced to carry relevant information to improve calibration performance. Among the monosensor approaches, the extra trees regression has the best results in IQAir sensors ($PM_{2.5}$ and $PM_{10}$), significantly improving the results of MultivarLinear_MonoSensor in $R^{2}$ and RMSE metrics. However, in the case of AirBeam, the best monosensor models correspond to multivariate linear regression, with slighly better $R^{2}$ and similar RMSE values. A possible explanation for this is that this type of sensor may have a more linear behavior than those of IQAir, making the benefit of using nonlinear models, such as extra trees, not evident.

For the multisensor models, the ensemble approach for $PM_{2.5}$ sensor calibration (Ensemb(ET)_MultiSensor) outperforms the merge approach in both performance indices. In the case of $PM_{10}$ sensors, the merge models tend to offer similar RMSE than Ensemb(ET)_MultiSensor models with some few cases also presenting better $R^{2}$ (AB2, AB3).

For the ensemble models that combine acknowledge period data and base models predictions (Ensemb(ET)_MultiSensor+Ack), we see better RMSE values compared to the ensemble model without acknowledge data in most cases, conserving the $R^{2}$ or improving it (HC3-$PM_{2.5}$, HC4-$PM_{2.5}$, HC3-$PM_{10}$, AB2-$PM_{2.5}$, AB3-$PM_{2.5}$, AB1-$PM_{10}$). The case of the IQAir $PM_{10}$ sensor HC1 clearly shows the advantage of using the acknowledge period, where the merge and ensemble models without acknowledge data present an RMSE of around 50 (close to the RMSE of the original data). However, the acknowledge period data leads the ensemble model performance to RMSE values less than eight. Most of the Ensemb(ET)_MultiSensor+Ack models present performances close to the best monosensor models. However, it is worth mentioning that these models only use a small proportion of field data from the target sensor (4 days) compared to the 40 days of field data used by the monosensor models. This implies that a new sensor can be incorporated into the monitoring network without needing long-period field data to fit its calibration function.

### 3.3. Data Quality Objectives

**Figure 12:**
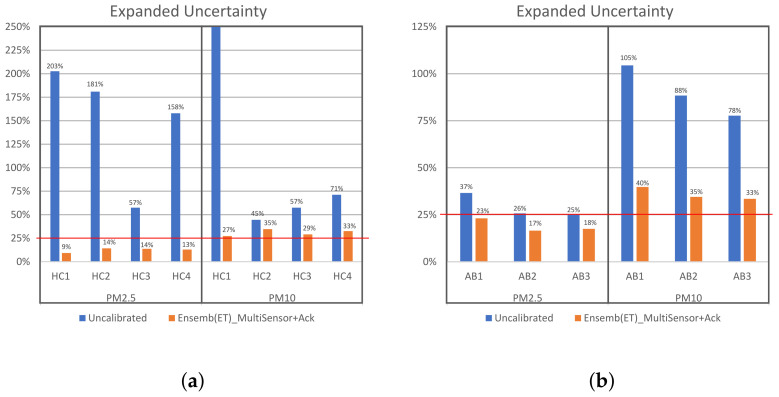
Expanded uncertainty of the original test measurements and of the data corrected by our best calibration models (Ensemb(ET)_MultiSensor+Ack) of the different IQAir and AirBeam sensors. The red line indicates the quality objective of 25% of expanded uncertainty to be considered as equivalent measurement for the monitoring of PM, according to Directive 2008/50/EC of the European Commission; 50% of expanded uncertainty is the quality objective for indicative measurements; (**a**) IQAir; (**b**) AIRBeam.

Here, we present an analysis of data quality objectives according to the Ambient Air Quality Directive 2008/50/EC, given by the European Commission (https://eur-lex.europa.eu/legal-content/en/ALL/?uri=CELEX%3A32008L0050, accessed on 10 December 2022). This is a kind of standard to assess the equivalence of non-reference measurement methods to the reference methods. To follow this guidance, we used the tool “Test the equivalence V3.1” to facilitate the use of the directive for PM monitoring. This is provided by the European Commission at https://circabc.europa.eu/ui/group/cd69a4b9-1a68-4d6c-9c48-77c0399f225d/library/24e15212-5858-4511-9da1-7ffb32683282/details (accessed on 10 December 2022). With this tool, we calculated the expanded uncertainty of the original measurements and of the data corrected by our best calibration models (Ensemb(ET)_MultiSensor+Ack). All the analyses were performed on the data of the test intervals. Figure 12 shows the expanded uncertainty (Wcm) on test data of the different IQAir and AirBeam sensors. It can be observed that in both pollutants ($PM_{2.5}$ and $PM_{10}$), the original measurements do not pass the quality objective of 25% of expanded uncertainty to be considered as equivalent measurement for the monitoring of particulate matter. The original measurements also fail to pass the 50% criterion to be considered as indicative measurement (except AirBeam $PM_{2.5}$ sensors and an IQAir $PM_{10}$ sensor). On the other hand, the corrected measurements of $PM_{2.5}$ by our best model exhibit an expanded uncertainty below the 25% criterion, so it can be considered an equivalent measurement. The corrected $PM_{10}$ measurements fail to pass the 25% criterion of expanded uncertainty, but in all cases, they exhibit an expanded uncertainty of less than 50%, so they can be considered as indicative measurements.

## 4. Conclusions

Currently, there is a strong interest in using low-cost technologies for air quality assessment in order to avoid poor air quality for citizens, deploy political strategies for public health and follow national and international regulations.

However, the data which arise from these sensors must be calibrated to ensure data quality. The fitting of calibration functions is usually performed ad hoc for each sensor to be incorporated in the network, requiring field data with a reference station for a period of time, which is an expensive and time-consuming process. In this article, we evaluated several approaches to generate calibration models for a new sensor for which we have no or very limited field data. The results lead us to conclude that the proposed strategy combining pre-fit calibration models on an ensemble of sensors together with a reduced period (4 days) of field data from the candidate sensor can provide performance similar to that of the best fitted monosensor model adjusted with field data from that sensor over a ten times longer period. With this approach, new sensors could be incorporated into a monitoring network quickly but still guaranteeing the quality of the data. A limitation of the study was the number of types of manufacturers and sensors. However, the results indicate that having field data from as little as two sensors is useful with the proposed approach to help build the calibration function for a new candidate sensor.

New research is needed to test the proposed approaches in different seasonal periods and possibly investigate new input variables, such as month or season of the year. The implications of our approach are the possibility of making available a multi-sensor calibration function that can serve as a pretrained model to conduct transfer learning to new sensors from the same manufacturer or from other manufacturers.

## Figures and Tables

**Table 1:** Cross-validated *RMSE* values of different monosensor calibration methods for calibrating $PM_{2.5}$ sensor values (IQAir and AirBeam). The cross-validation has been performed on training data using 10 folds. Values in parenthesis are the standard deviation of the *RMSE* values obtained in the 10 folds. Bold numbers indicate the best values in each row.

Manufacturer	Device	MultivarLinear	KNN	SVR	AdaBoost	GradientBoosting	Random Forest	Extra Trees
IQAir	HC1	2.55 (0.38)	2.33 (0.50)	2.87 (0.83)	2.99 (0.26)	2.16 (0.38)	2.24 (0.36)	**2.14 (0.29)**
	HC2	2.76 (0.47)	2.53 (0.60)	3.12 (1.01)	3.19 (0.39)	2.38 (0.26)	2.42 (0.35)	**2.29 (0.33)**
	HC3	2.51 (0.39)	2.23 (0.49)	2.85 (0.85)	2.86 (0.42)	2.19 (0.29)	2.16 (0.46)	**2.13 (0.35)**
	HC4	2.78 (0.48)	2.55 (0.52)	3.16 (0.83)	2.77 (0.38)	2.34 (0.28)	2.38 (0.38)	**2.34 (0.34)**
AirBEam	AB1	3.73 (0.40)	2.93 (0.33)	4.57 (0.79)	3.17 (0.29)	2.69 (0.23)	2.65 (0.27)	**2.65 (0.26)**
	AB2	3.74 (0.49)	2.84 (0.39)	4.33 (0.81)	3.17 (0.31)	2.73 (0.30)	**2.58 (0.35)**	2.62 (0.29)
	AB3	3.58 (0.65)	2.62 (0.41)	4.09 (0.85)	3.09 (0.39)	2.61 (0.38)	2.51 (0.36)	**2.48 (0.23)**

**Table 2:** Cross-validated *RMSE* values of different monosensor calibration methods for calibrating $PM_{10}$ sensor values (IQAir and AirBeam). The cross-validation has been performed on training data using 10 folds. Values in parenthesis are the standard deviation of the *RMSE* values obtained in the 10 folds. Bold numbers indicate the best values in each row.

Manufacturer	Device	LinearReg	KNN	SVR	AdaBoost	GradientBoosting	Random Forest	Extra Trees
IQAir	HC1	7.93 (0.95)	8.64 (1.06)	10.8 (1.51)	8.8 (0.91)	8.44 (1.14)	8.09 (1.07)	**8.07 (1.09)**
	HC2	7.92 (1.21)	8.08 (1.22)	9.98 (3.03)	8.74 (0.90)	8.26 (1.08)	7.95 (1.19)	**7.94 (1.11)**
	HC3	7.79 (1.00)	7.75 (1.09)	9.85 (1.76)	8.12 (0.83)	7.67 (0.97)	**7.38 (1.10)**	7.43 (0.83)
	HC4	8.47 (1.29)	8.59 (1.01)	10.3 (2.06)	9.01 (0.68)	8.86 (0.60)	**8.41 (0.71)**	8.53 (0.86)
AirBEam	AB1	10.2 (1.20)	9.47 (0.92)	11.4 (1.93)	9.64 (1.04)	10.1 (0.88)	9.5 (0.92)	**9.46 (1.03)**
	AB2	10.3 (0.82)	9.2 (0.77)	11 (1.73)	9.34 (0.68)	9.42 (0.71)	**9.05 (0.85)**	9.2 (0.83)
	AB3	9.84 (1.08)	8.87 (0.58)	10.8 (1.71)	9.21 (0.48)	8.94 (0.71)	8.74 (0.55)	**8.72 (0.56)**

## Data Availability

The datasets and Python codes to reproduce the results of this paper are made available at: shorturl.at/ftNQ4 (accessed on 4 April 2023).

## Abbreviations

The following abbreviations are used in this manuscript:

AQ	Air quality
AQI	Air quality index
ML	Machine learning
MLR	Multivariate linear regression
MSE	Mean squared error
RMSE	Root Mean squared error
PM	Particulate matter
$PM_{2.5}$	Particulate matter with aerosols of a maximum diameter of 2.5 μm
$PM_{10}$	Particulate matter with aerosols of a maximum diameter of 10 μm
